# Inhibition of Rumen Protozoa by Specific Inhibitors of Lysozyme and Peptidases *in vitro*

**DOI:** 10.3389/fmicb.2019.02822

**Published:** 2019-12-06

**Authors:** Tansol Park, Huiling Mao, Zhongtang Yu

**Affiliations:** ^1^Department of Animal Sciences, The Ohio State University, Columbus, OH, United States; ^2^College of Veterinary Medicine, Zhejiang A&F University, Lin’an, China

**Keywords:** rumen protozoa, nitrogen utilization efficiency, lysozyme, peptidases, inhibitors

## Abstract

Defaunation studies have shown that rumen protozoa are one of the main causes of low nitrogen utilization efficiency due to their bacterivory and subsequent intraruminal cycling of microbial protein in ruminants. In genomic and transcriptomic studies, we found that rumen protozoa expressed lysozymes and peptidases at high levels. We hypothesized that specific inhibition of lysozyme and peptidases could reduce the activity and growth of rumen protozoa, which can decrease their predation of microbes and proteolysis and subsequent ammoniagenesis by rumen microbiota. To test the above hypothesis, we evaluated three specific inhibitors: imidazole (IMI), a lysozyme inhibitor; phenylmethylsulphonyl fluoride (PMSF), a serine protease inhibitor; and iodoacetamide (IOD), a cysteine protease inhibitor; both individually and in combinations, with sodium dodecyl sulfate (SDS) as a positive control. Rumen fluid was collected from two Jersey dairy cows fed either a concentrate-based dairy ration or only alfalfa hay. Each protozoa-enriched rumen fluid was incubated for 24 h with or without the aforementioned inhibitors and fed a mixture of ground wheat grain, alfalfa, and grass hays to support microbial growth. Live protozoa cells were morphologically identified and counted simultaneously at 3, 6, 12, and 24 h of incubation. Fermentation characteristics and prokaryotic composition were determined and compared at the end of the incubation. Except for IOD, all the inhibitors reduced all the nine protozoal genera identified, but to different extents, in a time-dependent manner. IOD was the least inhibitory to protozoa, but it lowered ammoniagenesis the most while not decreasing feed digestibility or concentration of volatile fatty acids (VFA). ANCOM analysis identified loss of *Fibrobacter* and overgrowth of *Treponema*, *Streptococcus*, and *Succinivibrio* in several inhibitor treatments. Functional prediction (from 16S rRNA gene amplicon sequences) using the CowPI database showed that the inhibitors decreased the relative abundance of the genes encoding amino acid metabolism, especially peptidases, and lysosome in the rumen microbiota. Overall, inhibition of protozoa resulted in alteration of prokaryotic microbiota and *in vitro* fermentation, and peptidases, especially cysteine-peptidase, may be targeted to improve nitrogen utilization in ruminants.

## Introduction

Ruminants depend on the diverse rumen microbial assembly, comprising bacteria, archaea, protozoa, and fungi, for their survival and growth and the production of animal products (beef, lamb, milk, and wool). Collectively, the rumen microbial digestive and fermentative processes convert dietary carbohydrates, primarily starch and cellulose, and dietary nitrogen, primarily plant protein nitrogen, into the carbon and nitrogen sources that ruminants can utilize. The utilization efficiency of dietary nitrogen in ruminants is low, only about 25% (Kohn et al., 2005; Huhtanen and Hristov, 2009). Such a low nitrogen utilization efficiency not only increases the production cost but also creates environmental pollution with nitrogen. Indeed, of all the ammonia and nitrous oxide released into the environment by anthropogenic activities, about 70 and 30%, respectively, were estimated to stem from livestock husbandry (Aardenne et al., 2001). Approximately 70% of the dietary nitrogen (primarily as protein) is hydrolyzed in the rumen to oligopeptides and free amino acids, both of which are fermented to short-chain fatty acid (SCFA) and ammonia. Some of the ammonia nitrogen (NH_3_-N) is used as the nitrogen sources for ruminal microbes, primarily bacteria, to synthesize cellular proteins, which are the major direct nitrogen source for the host animals (Storm et al., 1983; Leng and Nolan, 1984; Hackmann and Firkins, 2015), but a large portion of the microbial cells (about 24% of the total ruminal bacteria daily) are engulfed by ruminal protozoa (Hespell et al., 1997), and approximately 50% of the engulfed bacterial proteins is hydrolyzed by protozoa to form oligopeptides and free amino acids (Jouany, 1996), which can be fermented back to SCFA and ammonia. Although varying in ability and rate (Belanche et al., 2012), all protozoa engulf microbial cells, even the small protozoa, and subsequently degrade the microbial proteins to oligopeptides and free amino acids (Williams and Coleman, 1992). Thus, ruminal protozoa mediate intraruminal recycling of microbial protein and consequently decrease the ruminal outflow of microbial proteins.

Ruminal protozoa have been considered as a non-vital group of microbes for the host animals even though they contribute to feed digestion and homeostasis of the rumen environment (Newbold et al., 2015). Indeed, elimination of rumen protozoa (i.e., defaunation) was shown to have little effect on feed digestion or fermentation but increase dietary nitrogen utilization efficiency (Belanche et al., 2011) and decrease methane emission (Newbold et al., 2015). However, defaunation is infeasible at the farm level and can be inimical to animals, such as decreased feed intake and digestion (Eugène et al., 2004; Newbold et al., 2015). Therefore, numerous studies have attempted to control ruminal protozoa using plant extracts or lipids (Wang et al., 2000; Hu et al., 2005; Patra and Yu, 2012, 2015; Patra et al., 2012), but none of them could achieve consistent inhibition of rumen protozoa. Protozoa depend on both lysozyme and peptidases to lyse and digest the engulfed microbial cells (Bonhomme, 1990; Morgavi et al., 1996). Indeed, the macronuclear genome (the transcriptionally active genome) of *Entodinium caudatum* carried multiple genes encoding both lysozymes and different families of peptidases (Park et al., 2018). These genes are also highly expressed in actively growing monocultures of *E*. *caudatum* (unpublished data).

We hypothesized that specific inhibition of lysozyme and peptidases could reduce the activity and growth of rumen protozoa, which would decrease their predation on microbial cells and proteolysis and subsequent ammoniagenesis by rumen microbiota, with little or no adverse collateral effect on feed digestion or fermentation. The above hypothesis has been tested using a monoculture of *E. caudatum* that had been maintained in laboratory (Park et al., 2019). The objective of this study was to test the above hypothesis using fresh rumen fluid that contains all the rumen protozoa and other microbes typically found in the rumen. We evaluated three specific inhibitors *in vitro*: imidazole (IMI, a specific lysozyme inhibitor), phenylmethylsulphonyl fluoride (PMSF, a specific serine protease inhibitor), and iodoacetamide (IOD, a specific cysteine protease inhibitor), both individually and in two- or three-way combinations, with sodium dodecyl sulfate (SDS) serving as a positive control for defaunation. We also used rumen fluid from both lactating cows fed a typical dairy ration and non-lactating cows fed only alfalfa hay to representing the two stages of dairy production. The effects of the inhibitors on feed digestion, rumen fermentation, and the prokaryotic microbiota were also examined to determine the potential adverse effects.

## Materials and Methods

### Inhibitor Selection

In a recent study, known specific inhibitors of lysozyme, cysteine peptidases, serine peptidases, and metallopeptidases were screened using a monoculture of *E. caudatum*, and IMI (inhibiting lysozyme) at 100 mmol/L, PMSF (inhibiting serine peptidases) at 3 mmol/L, and IOD (inhibiting cysteine peptidases) at 0.5 mmol/L were found effective in inhibiting *E*. *caudatum* and lowering ammonia concentration without decreasing feed digestion or fermentation (Park et al., 2019). In the present study, those three inhibitors at the above concentrations were further evaluated with rumen fluid containing all the diverse protozoal and other microbial species typically found in the rumen of dairy cows. SDS, which was effective in achieving *in vitro* defaunation of rumen microbiota (Qin et al., 2012), was included at 1.44 mmol/L as a positive control for defaunation. A stock solution of each inhibitor was prepared aseptically in water, except for PMSF, which is insoluble in water, that was dissolved in absolute ethanol. One control containing none of the inhibitors but water (referred to as water control) was included. One control containing the same amount of ethanol (referred to as ethanol control) as the PMSF treatment was also included. This ethanol control was excluded from further analysis because it did not significantly alter protozoa counts, feed digestion, fermentation, ammonia concentration, or microbiota composition as compared to the water control (data not shown).

### Preparation of Protozoa-Enriched Rumen Fluid and *in vitro* Experimental Procedures

Fresh rumen fluid was collected from two rumen-cannulated Jersey cows, with one being fed a concentrate-based dairy ration typical for lactating cows and the other being fed alfalfa hay only. Rumen fluid was collected 2 h after morning feeding and kept warm in tightly closed bottles during transfer (less than 15 min) to laboratory. The rumen fluid samples were left still in sealed bottles and kept at 39°C for 1 h in a water bath to allow protozoa to settle and concentrate. Then, the supernatant was carefully removed without disturbing the middle or the bottom phases. A continuous CO_2_ stream flushed the headspace of each bottle to prevent exposure of the protozoa-enriched rumen fluid to the air.

The setup of the *in vitro* cultures was the same as that reported previously (Park et al., 2019). Briefly, 10 ml of protozoa-enriched rumen fluid were each inoculated into one 120-ml serum bottle containing 20 ml of artificial medium (Goering and Van Soest, 1970) and 0.3 g of the protozoa feed (0.1 g each of ground wheat, alfalfa, and grass), and the inhibitor at the pre-set concentrations. Each inhibitor was evaluated using four *in vitro* cultures (four replicates) inoculated with the protozoa-enriched inoculum from the two donor cows. Blanks without substrates and control without inhibitors were included to aid determination of dry matter (DM) and neutral detergent fiber (NDF) present in the rumen fluid inoculum along with the protozoal counts at the beginning of the experiment. All the cultivation procedures were done under anaerobic conditions maintained using continuous O_2_-free CO_2_ flushing. Each *in vitro* culture was subsampled (0.5 ml) at 3, 6, 12, and 24 h of incubation for enumeration and differentiation of protozoa using microscopy as done previously (Park et al., 2017). Briefly, each subsample was fixed with a fixative containing 16.67% formalin, 10% glycerol, and brilliant green dye to preserve and stain the protozoal cells. Cells of each morphologically identified protozoan genus were counted using a counting chamber (Hausser Scientific, catalog #3800) under a microscope at 100× magnification (Dehority, 1998).

After 24 h incubation, 2 ml of cultures were collected into a 2-ml microtube and centrifuged at 16,000 × *g* at 4°C for 10 min. The resulting pellets representing the total microbiota were preserved in −80°C until DNA extraction. The supernatants were divided into two aliquots, with one being stored at −20°C for analysis of ammonia concentration, while the other being mixed with one volume of 33% metaphosphoric acid and filtered for analysis of volatile fatty acids (VFA). The rest of the cultures (approximately 26 ml) were each filtered through an Ankom fiber filter bag (50 μm pore size) and dried at 55°C for 48 h to determine DM digestibility (DMD) followed by subsequent determination of NDF digestibility (NDFD) (Van Soest et al., 1991). The concentrations of ammonia were determined using a colorimetric method (Chaney and Marbach, 1962), and VFA concentrations were determined using gas chromatography (Pantoja et al., 1994).

### DNA Extraction and Microbiome Analysis

Metagenomic DNA from each *in vitro* culture pellet was extracted using the RBB + C method (Yu and Morrison, 2004). Only three of the four replicates were used in the DNA extraction due to the loss of one replicate in one of the treatments. The quality and quantity of the extracted DNA were assessed based on 260/280 and 260/230 ratios determined using a NanoDrop ND-2000 Spectrophotometer (Thermo Scientific, NanoDrop Technologies, Wilmington, DE, United States) followed by agarose gel (1%, w/v) electrophoresis. The prokaryotic microbiota of each sample was analyzed using 16S rRNA gene amplicon sequencing as done previously (Park et al., 2019). Briefly, one amplicon library was prepared using PCR amplification of the V4 hypervariable region of 16S rRNA genes using the primer set 515F and 806R (Caporaso et al., 2010) with each amplicon library having a unique barcode for multiplexing. The amplicon libraries were pooled at an equal ratio and sequenced using the 2 × 300 paired-end protocol on the Illumina MiSeq platform. 16S amplicon sequences have been deposited in the NCBI Sequence Read Archive (SRA) under BioProject PRJNA523838.

The amplicon sequences were first analyzed using the built-in commands and plugins within QIIME2 (Bolyen et al., 2018). Briefly, after adapter sequence removal using Cutadapt (Martin, 2011), the demultiplexed paired-end reads were quality-filtered (Q > 25), denoised, merged, and potential chimeric sequences were filtered out using the DADA2 plugin (Callahan et al., 2016). Amplicon sequencing variants (ASVs) were clustered at 99% similarity using the Greengenes 16S reference database (13_8 version) (McDonald et al., 2012), which was manually trained based on the targeted V4 hypervariable region using the Naïve Bayes classifier (Bokulich et al., 2018). Major phyla and genera each representing >0.5% of total sequences on average across all the samples were discussed in this study. Alpha-diversity measurements including species richness, evenness, Faith’s phylogenetic diversity, and Shannon’s diversity index were calculated based on the rarefied ASV tables using 16,614 sequences per sample (Supplementary Table S1). Richness was calculated at the genus level. Principal coordinates analysis (PCoA) based on weighted UniFrac distances was used to compare the overall dissimilarity of microbiota shaped by the inhibitors. Metabolic functions were predicted using the CowPI database, a rumen microbiome-focused version of the PICRUSt (Wilkinson et al., 2018) from the OTUs picked using the closed-reference approach against the Greengenes 13_8 97% OTUs reference database. The overall dissimilarity of the functional features among the *in vitro* cultures was analyzed using principal component analysis (PCA) based on Bray-Curtis dissimilarity. Co-occurrence and mutual exclusion networks were generated based on the Pearson correlation coefficients between the major genera (each having a relative abundance ≥ 0.5% in both treatments) and visualized using Gephi (Bastian et al., 2009). Only significant relationships with a *P*-value adjusted with Benjamini-Hochberg correction (Benjamini and Hochberg, 1995) below 0.05 were shown in the networks.

### Quantitative Real-Time PCR

The absolute abundance of total bacteria and archaea in each sample was quantified as 16S rRNA gene copies per ml sample using quantitative real-time PCR with each domain-specific primer set (340f/806r for bacteria, and Met86f/Met915r for archaea) (Nadkarni et al., 2002; Wright and Pimm, 2003; Watanabe et al., 2004) as done in our previous studies (Stiverson et al., 2011; Park and Yu, 2018b). The relative abundance of total archaea was calculated as a percent of total prokaryotic 16S rRNA gene copies.

### Statistical Analysis

The two experimental runs using the rumen fluid samples collected from the cows fed the two different diets were combined for the statistical analysis. Rumen protozoal counts of identified genera were log_10_-transformed followed by analysis using the GLIMMIX procedure of SAS 9.4 (SAS Institute Inc., Cary, NC, United States). To account for the repeated measurements over time of incubation, we added “Time” in the repeated-measures statement within an *in vitro* culture with unstructured variance-covariance structure. The statistical model included the fixed effects of treatments (inhibitors), diets (dairy ration or hay), and incubation times. Interaction between treatment and diet was also included in the model for all the tested variables. Orthogonal contrasts were used to analyze the effects of the incubation times within each inhibitor on the protozoal growth. The treatment effects on the fermentation characteristics (DMD, NDFD, ammonia concentration, pH, and VFA profiles), the absolute abundance of total bacteria and archaea, and alpha diversity measurements were also analyzed using SAS 9.4. Differences were assessed using Tukey’s honest significance test, and significance was declared at *P* ≤ 0.05 and tendency at 0.05 < *P* ≤ 0.10. Permutational multivariate analysis of variance (PERMANOVA) was used to assess the PCoA and PCA plots using the PAST3 software with 9,999 random permutations (Hammer et al., 2001). Differentially predominant taxa between the control and each treatment were identified using the ANCOM test (Mandal et al., 2015) implemented in QIIME2 with BIOM tables (collapsed at phylum and genus levels) as the input. Pearson correlation coefficients among protozoal counts, relative abundance of major phyla and genera of prokaryotes and fermentation characteristics, predicted functions related to protein metabolism were calculated using PROC CORR procedure of SAS 9.4 and visualized using R package corrplot (v. 3.5.0).

## Results

### The Protozoa Present in the Rumen of the Two Cows

The initial protozoal counts reached 1.60 × 10^5^ and 4.67 × 10^4^ cells per ml of *in vitro* cultures of the dairy ration-fed and the hay-fed donors, respectively. In total eight genera of protozoa were found in the rumen of both cows. *Entodinium* was the most dominant genus, accounting for >86% of total protozoa in the rumen of both cows, followed with other genera, including *Dasytricha* and *Isotricha*, at much lower relative abundance.

### Effects of Lysozyme and Peptidase Inhibitors on the Counts of Rumen Protozoa

Total protozoa counts were significantly decreased by all the inhibitors except IOD in a time-dependent manner (Table 1). However, different genera appeared to be inhibited to different extents, with *Entodinium* being less inhibited than the other genera. Among the three inhibitors, the lysozyme inhibitor IMI inhibited the identified rumen protozoa the most, while the cysteine peptidase inhibitor IOD led to the least inhibition. Overall, the combinations, both two- and three-way, inhibited all the identified genera of protozoa to a greater extent than the inhibitors individually. All the protozoal genera were completely inhibited by SDS by 12 h of incubation.

**TABLE 1:** Inhibition of the identified protozoal genera (log-transformed counts) by the inhibitors (at 3, 6, 12, and 24 h of incubation).

**Protozoa genera**	**Treatments^1^**										**SEM**	**Effects (*P*-values)**		
			
	**Time**	**Control**	**IMI**	**PMSF**	**IOD**	**IMI-PMSF**	**IMI-IOD**	**PMSF-IOD**	**3Mix^2^**	**SDS**		**Trt^3^**	**Time**	**Diet**
Total protozoa														
	3 h	5.12^A^	5.04^A^	5.06^A^	5.05^A^	4.79^B^	4.99^A^	5.02^A^	4.73^B^	1.50^C^	0.15	<0.0001	0.0334	0.0329
	6 h	5.14^A^	4.94^A^	5.02^A^	5.04^A^	4.35^BC^	4.84^AB^	4.78^AB^	4.11^C^	0.31^D^	0.18			
	12 h	5.16^A^	4.59^AB^	4.94^AB^	5.01^AB^	3.37^C^	4.45^B^	4.50^B^	3.12^C^	0^D^	0.20			
	24 h	5.13^A^	2.97^C^	4.36^AB^	4.80^A^	0^D^	2.83^C^	3.69^BC^	0^D^	0^D^	0.25			
	Contrast (time)^∗^	–	L,Q	L	–	L,Q	L	L	L	L,Q,C				
*Entodinium*														
	3 h	5.01^A^	4.94^A^	4.94^A^	4.93^A^	4.77^A^	4.89^A^	4.90^A^	4.67^A^	1.06^B^	0.16	<0.0001	0.0320	0.0107
	6 h	5.04^A^	4.89^AB^	4.90^AB^	4.94^AB^	4.35^D^	4.80^BC^	4.67^C^	4.10^E^	0^D^	0.19			
	12 h	5.06^A^	4.57^AB^	4.86^AB^	4.88^AB^	3.37^C^	4.44^AB^	4.42^B^	3.12^C^	0^D^	0.19			
	24 h	5.06^A^	2.97^C^	4.32^AB^	4.69^A^	0^D^	2.83^C^	3.69^BC^	0^D^	0^D^	0.25			
	Contrast (time)^∗^	–	L,Q	L	–	L,Q	L,Q	L	L,Q	L,Q,C				
*Diplodinium*														
	3 h	3.16^A^	2.72^A^	3.22^A^	3.14^A^	1.64^B^	2.62^A^	3.07^A^	2.31^AB^	0^C^	0.16	<0.0001	0.0036	<0.0001
	6 h	3.05^A^	1.99^A^	2.69^A^	2.68^A^	0.62^BC^	2.23^A^	1.86^AB^	0.31^C^	0^C^	0.18			
	12 h	3.19^A^	0.69^BC^	2.61^A^	2.78^A^	0^C^	0.96^BC^	1.47^B^	0^C^	0^C^	0.18			
	24 h	2.97^A^	0^C^	1.20^B^	2.59^A^	0^C^	0^C^	0^C^	0^C^	0^C^	0.15			
	Contrast (time)^∗^	–	L	L	–	L,Q	L	L	L,Q,Qt	L,Q,C,Qt				
*Isotricha*														
	3 h	3.61^A^	3.42^A^	3.51^A^	3.53^A^	2.50^B^	3.34^A^	3.57^A^	1.47^C^	0^D^	0.16	<0.0001	<0.0001	0.6990
	6 h	3.58^A^	3.04^A^	3.49^A^	3.49^A^	0.96^B^	2.65^A^	3.43^A^	0.31^BC^	0^C^	0.18			
	12 h	3.52^A^	0.64^BC^	3.35^A^	3.45^A^	0^C^	1.01^B^	3.14^A^	0^C^	0^C^	0.20			
	24 h	3.53^A^	0^C^	2.31^B^	3.16^A^	0^C^	0^C^	0.31^CD^	0^C^	0^C^	0.18			
	Contrast (time)^∗^	C	L,Q,C	L	L	L,Q	L,C	L,Q	L,Q,C	L,Q,C,Qt				
*Dasytricha*														
	3 h	3.66^A^	2.37^B^	3.63^A^	3.49^A^	0.64^C^	2.60^AB^	3.52^A^	0.64^C^	0^C^	0.20	<0.0001	0.1786	0.0039
	6 h	3.58^A^	1.73^B^	3.56^A^	3.62^A^	0^C^	1.85^B^	3.25^A^	0^C^	0^C^	0.20			
	12 h	3.69^A^	0^C^	3.37^A^	3.71^A^	0^C^	0^C^	2.27^B^	0^C^	0^C^	0.21			
	24 h	3.60^A^	0^D^	2.06^B^	3.68^A^	0^D^	0^D^	0.98^C^	0^D^	0^D^	0.19			
	Contrast (time)^∗^	–	L,Q	L	–	L,Q,C	L,Q	L	L,Q,C	L,Q,C,Qt				
*Epidinium*														
	3 h	2.07^AB^	1.87^AB^	3.11^A^	2.60^A^	1.86^AB^	2.15^AB^	2.34^A^	2.50^A^	0.62^B^	0.17	<0.0001	0.0538	0.0246
	6 h	2.95^A^	2.89^A^	3.17^A^	2.86^A^	0.65^B^	2.09^A^	2.32^A^	0.65^B^	0^B^	0.17			
	12 h	3.03^A^	0.61^BC^	2.94^A^	2.52^A^	0^C^	0.55^BC^	1.30^B^	0^C^	0^C^	0.17			
	24 h	2.39^A^	0^B^	0.31^B^	2.01^A^	0^B^	0^B^	0^B^	0^B^	0^B^	0.13			
	Contrast (time)^∗^	–	L,Qt	L,Q	–	L,Q	L	L	L,Q,Qt	L,Q,C				
*Diploplastron*														
	3 h	3.13^A^	3.12^A^	2.96^A^	3.18^A^	2.64^A^	3.11^A^	3.01^A^	2.98^A^	1.04^B^	0.10	<0.0001	0.0003	0.0020
	6 h	3.13^A^	2.88^A^	2.98^A^	2.77^A^	1.44^B^	2.90^A^	2.84^A^	0.71^BC^	0.31^C^	0.15			
	12 h	3.21^A^	1.94^BC^	2.85^AB^	3.17^A^	0^D^	1.62^C^	1.75^C^	0^D^	0^D^	0.17			
	24 h	3.10^A^	0^B^	0.65^B^	2.92^A^	0^B^	0^B^	0^B^	0^B^	0^B^	0.15			
	Contrast (time)^∗^	Q	L,Q	L,Q	–	L,Q	L,C	L	L,Q,Qt	L,Q,C				
*Ophryoscolex*														
	3 h	3.08^A^	2.74^A^	2.64^A^	2.64^A^	1.94^A^	2.61^A^	2.95^A^	2.22^A^	0^B^	0.16	<0.0001	0.0002	<0.0001
	6 h	3.04^A^	2.57^AB^	3.09^A^	2.92^A^	0^D^	2.27^ABC^	1.74^BC^	1.24^C^	0^D^	0.17			
	12 h	2.94^A^	1.69^ABC^	2.15^AB^	2.78^A^	0^D^	1.37^BCD^	0.31^CD^	0^D^	0^D^	0.17			
	24 h	2.99^A^	0^B^	0.30^B^	0.66^B^	0^B^	0^B^	0^B^	0^B^	0^B^	0.12			
	Contrast (time)^∗^	–	L	L	L,Q	L,Q,Qt	L	L,Q,C	L,Q	L,Q,C,Qt				
*Polyplastron*														
	3 h	2.81^A^	1.57^ABC^	2.35^AB^	2.43^A^	0.62^C^	1.24^ABC^	1.09^ABC^	0.66^BC^	0^C^	0.16	<0.0001	0.0909	0.6485
	6 h	2.70^A^	0.91^BC^	1.94^AB^	2.39^AB^	0^C^	0.95^BC^	1.28^ABC^	0^C^	0^C^	0.16			
	12 h	1.99^A^	0^B^	0.99^AB^	2.08^A^	0^B^	0.55^B^	0.68^AB^	0^B^	0^B^	0.14			
	24 h	2.52^A^	0^C^	0^C^	1.24^B^	0^C^	0^C^	0^C^	0^C^	0^C^	0.11			
	Contrast (time)^∗^	C	L,Q	L	L	L,Q,C	L	L	L,Q,C	L,Q,C,Qt				

*^1^IMI, imidazole at 100 mmol/L; PMSF, phenylmethylsulphonyl fluoride at 3 mmol/L; IOD, iodoacetamide at 0.5 mmol/L; SDS, sodium dodecyl sulfate at 1.44 mmol/L; ^2^3Mix, combination of IMI, PMSF, and IOD at the above concentrations; ^3^Treatment. Means (*n* = 8) followed by different superscripts in a row differ significantly (*P* < 0.05). ^∗^Contrast: *P*-value < 0.05 was considered as significant. L, linear; Q, quadratic; C, cubic; and Qt, quartic effect of time. Interaction between Trt and Diet was significant (*P* < 0.05) except for total protozoa and *Ophryoscolex* (*P* > 0.1).*

### Effect of the Lysozyme and Peptidase Inhibitors on the Prokaryotic Microbiota

**FIGURE 1:**
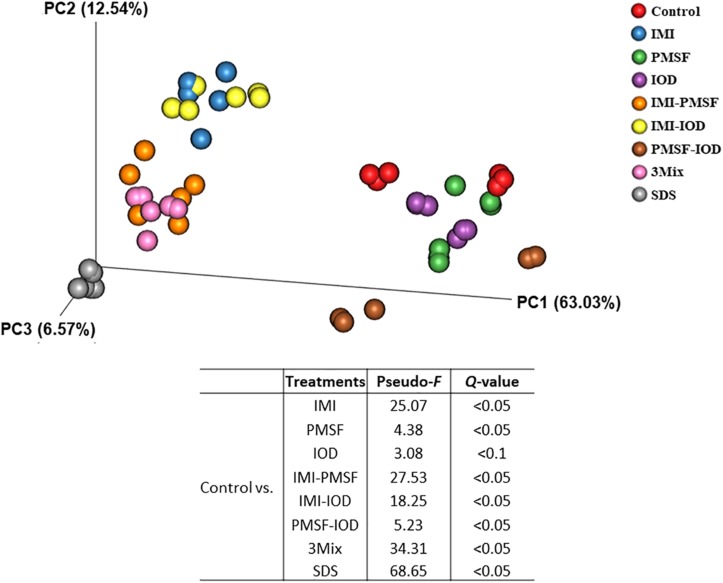
Principal coordinates analysis based on the weighted UniFrac distance matrices of the prokaryotic microbiota in the *in vitro* cultures. PERMANOVA results of the pairwise comparison of the overall microbiota structures between control and each of inhibitor treatment was included.

The prokaryotic microbiota was examined and compared among the treatments in both alpha and beta diversity measurements. At least 16,614 sequences were obtained from each of the samples (Supplementary Table S1), allowing for a Good’s coverage > 99.3% for all the samples (data not shown). All the inhibitor treatments significantly decreased the number of observed genera and Faith’s phylogenetic diversity (Table 2). The evenness was decreased in all the treatments except PMSF and IOD. The IMI treatment significantly lowered all diversity measurements except Chao1 estimate. All the two- and three-way combinations decreased the richness (both observed and Chao1 estimate), evenness, Faith’s phylogenetic diversity, and Shannon diversity index. The SDS treatment decreased all alpha diversity measurements significantly. The PCoA based on weighted UniFrac distances showed that all the inhibitors except IOD significantly shift the overall microbiota composition compared to the control (Figure 1). The SDS treatment affected the prokaryotic microbiota the most.

**TABLE 2:** Alpha-diversity measurements of the microbiota (at 24 h of incubation).

**Treatments^1^**	**Observed genera**	**Chao1 (Genera)**	**Evenness**	**PD^2^ index**	**Shannon’s**
Control	88^A^	90^A^	0.80^A^	42.99^A^	7.71^A^
IMI	79^B^	80^AB^	0.62^E^	31.67^B^	5.41^D^
PMSF	75^B^	77^B^	0.80^AB^	33.75^B^	7.29^AB^
IOD	63^C^	63^C^	0.79^AB^	28.61^BC^	6.92^B^
IMI-PMSF	73^B^	74^B^	0.62^DE^	28.57^BC^	5.31^D^
IMI-IOD	76^B^	76^B^	0.72^BC^	30.10^BC^	6.18^C^
PMSF-IOD	58^CD^	59^C^	0.70^CD^	25.92^C^	5.83^CD^
3Mix^3^	74^B^	75^B^	0.60^E^	28.99^BC^	5.14^D^
SDS	52^D^	52^C^	0.51^F^	15.51^D^	3.62^E^
SEM	1.67	1.79	0.02	1.09	0.18
Trt^4^	<0.0001	<0.0001	<0.0001	<0.0001	<0.0001
Diet	0.0006	0.0048	0.0032	0.0241	<0.0001
T × D	<0.0001	0.0002	<0.0001	<0.0001	<0.0001

*^1^IMI, imidazole at 100 mmol/L; PMSF, phenylmethylsulphonyl fluoride at 3 mmol/L; IOD, iodoacetamide at 0.5 mmol/L; SDS, sodium dodecyl sulfate at 1.44 mmol/L; ^2^Faith’s phylogenetic diversity; ^3^3Mix, combination of IMI, PMSF, and IOD at the above concentrations; ^4^Treatment. Means (*n* = 6) followed by different superscripts in a column differ significantly (*P* < 0.05).*

Statistical analysis using ANCOM identified major bacterial phyla and genera that were affected by the inhibitor treatments when compared to the control (Table 3). *Spirochetes* was increased by PMSF, IOD, and their combination but decreased by IMI-IOD and SDS. The genus *Fibrobacter* almost disappeared in the IMI-containing treatments and the SDS treatment (Table 3), but its relative abundance was increased by 2.60-fold by IOD (Supplementary Figure S1). *Streptococcus* was increased by IMI, IMI-IOD, and SDS. A sharp increase of *Succinivibrio* was observed in the IMI-PMSF, PMSF-IOD, and 3Mix treatments. *Acidaminococcus* was found in all the IMI-containing treatments and SDS treatment but not in the control, and the relative abundance of *Ruminobacter* was increased by all the treatments up to 35.43% (Supplementary Figure S1). *Treponema*, the representative genus of *Spirochetes*, which had a relative abundance at 0.53% in the control, expanded its relative abundance, reaching about 12% in the PMSF and PMSF-IOD treatments (Supplementary Figure S1 and Table 3).

**TABLE 3:** Major differentially abundant bacterial phyla and genera (each representing >0.5% of total sequences in at least one of the treatments) defined by ANCOM between the controls and each inhibitor treatment.

**Taxa**	**Treatments^1^**	**Relative abundance, %(Control vs. Trt^2^)**	**Mean difference (log)**
**Phylum**			
*Fibrobacteres*	IMI	(1.894 vs. 0.004)	–6.680
*Spirochetes*	PMSF	(0.844 vs. 11.942)	2.195
*Fibrobacteres*	IOD	(1.894 vs. 4.931)	0.422
*Spirochetes*	IOD	(0.844 vs. 2.377)	0.612
*Fibrobacteres*	IMI-PMSF	(1.894 vs. 0)	–6.979
*Fibrobacteres*	IMI-IOD	(1.894 vs. 0)	–6.979
*Spirochetes*	IMI-IOD	(0.844 vs. 0.090)	–2.833
*Spirochetes*	PMSF-IOD	(0.844 vs. 11.919)	1.710
*Fibrobacteres*	3Mix^3^	(1.894 vs. 0)	–6.796
*Fibrobacteres*	SDS	(1.894 vs. 0)	–6.979
*Spirochetes*	SDS	(0.844 vs. 0)	–5.779
*Proteobacteria*	SDS	(17.547 vs. 67.556)	1.566
**Genera**			
*Fibrobacter*	IMI	(1.894 vs. 0.004)	–6.680
*Streptococcus*	IMI	(0.003 vs. 0.977)	5.661
*Treponema*	PMSF	(0.525 vs. 11.896)	2.667
*Pseudobutyrivibrio*	IOD	(0.114 vs. 0.635)	1.247
*Treponema*	IOD	(0.525 vs. 2.133)	0.989
*Fibrobacter*	IMI-PMSF	(1.894 vs. 0)	–6.979
*Succinivibrio*	IMI-PMSF	(0.396 vs. 37.405)	4.630
*Fibrobacter*	IMI-IOD	(1.894 vs. 0)	–6.979
*Streptococcus*	IMI-IOD	(0.003 vs. 4.001)	6.275
*Succinivibrio*	PMSF-IOD	(0.396 vs. 19.505)	3.807
*Shuttleworthia*	PMSF-IOD	(0.058 vs. 0.868)	2.226
*Fibrobacter*	3Mix	(1.894 vs. 0)	–6.796
*Succinivibrio*	3Mix	(0.396 vs. 43.083)	4.822
*Fibrobacter*	SDS	(1.894 vs. 0)	–6.979
*Streptococcus*	SDS	(0.003 vs. 1.732)	5.721

*^1^IMI, imidazole at 100 mmol/L; PMSF, phenylmethylsulphonyl fluoride at 3 mmol/L; IOD, iodoacetamide at 0.5 mmol/L; SDS, sodium dodecyl sulfate at 1.44 mmol/L; ^2^Treatment; ^3^3Mix, combination of IMI, PMSF, and IOD at the above concentrations.*

Co-occurrence and mutual-exclusion network analyses showed altered relationships between major genera in response to each single inhibitor treatment (Supplementary Figure S2). Individual inhibitor treatments lowered prokaryotic microbial co-occurrence complexity compared to that of control based on the number of nodes, edges, contribution to the total community.

### Effects of Lysozyme and Peptidase Inhibitors on Fermentation Characteristics

Compared to the control, the lysozyme inhibitor IMI, its combinatorial treatments, and SDS lowered DMD and NDFD (Table 4). PMSF and IOD treatments maintained DMD and NDFD but their combination lowered NDFD. IOD, IMI-PMSF, PMSF-IOD, and 3Mix reduced ammonia concentration. Additive inhibition to ammonia concentration was not noted. The IMI-containing treatments increased the pH significantly, while only PMSF lowered the pH. Total bacteria was not differed by the inhibitor treatments except IOD and IMI-IOD, but SDS reduced both the absolute and relative abundances of methanogens.

**TABLE 4:** Digestibility, fermentation characteristics, and abundance of total bacteria and archaea (log-copies of 16S rRNA genes per ml of sample) at 24 h of incubation.

	**Treatments^1^**									**SEM**	**Effects (*P*-values)**		
			
	**Control**	**IMI**	**PMSF**	**IOD**	**IMI-PMSF**	**IMI-IOD**	**PMSF-IOD**	**3Mix^2^**	**SDS**		**Trt^3^**	**Diet**	**T × D**
DM digestibility, %	76.57^A^	66.57^B^	78.51^A^	76.80^A^	62.13^C^	65.00^BC^	75.06^A^	62.50^C^	68.18^B^	0.85	<0.0001	0.0008	<0.0001
NDF digestibility, %	77.67^A^	57.61^C^	76.53^A^	76.33^A^	57.31^C^	57.37^C^	72.21^B^	57.42^C^	59.36^C^	1.11	<0.0001	0.0280	<0.0001
NH_3_-N, mg/dL	13.91^AB^	14.76^A^	10.60^BC^	5.63^D^	8.82^CD^	11.38^ABC^	6.14^D^	6.69^D^	12.56^AB^	0.48	<0.0001	0.0460	0.0005
pH	6.45^BC^	7.15^A^	6.37^D^	6.45^B^	7.09^A^	7.12^A^	6.38^CD^	7.10^A^	6.43^BCD^	0.04	<0.0001	<0.0001	0.0004
Total VFA (mM)	79.68^A^	73.48^BC^	77.65^AB^	74.78^ABC^	65.15^DE^	69.64^CD^	69.47^CD^	61.06^E^	69.62^CD^	1.96	<0.0001	<0.0001	<0.0001
Acetate, %	60.41^B^	61.69^A^	59.35^C^	53.24^F^	61.50^A^	58.15^D^	53.10^F^	57.95^D^	56.60^E^	0.54	<0.0001	<0.0001	<0.0001
Propionate, %	17.57^F^	20.65^E^	20.37^E^	24.23^BC^	22.82^D^	23.50^CD^	27.44^A^	24.70^B^	27.50^A^	0.40	<0.0001	<0.0001	<0.0001
Butyrate, %	16.52^B^	11.80^EF^	14.90^C^	17.18^A^	11.43^F^	12.31^D^	14.97^C^	12.07^DE^	10.23^G^	0.52	<0.0001	<0.0001	<0.0001
Valerate, %	2.08^BC^	2.00^C^	2.11^BC^	2.48^A^	1.61^D^	2.26^AB^	2.13^BC^	1.97^C^	2.11^BC^	0.04	<0.0001	0.1107	<0.0001
BCVFA^4^, %	3.42^ABC^	3.86^A^	3.26^CD^	2.87^DE^	2.65^EF^	3.78^AB^	2.37^F^	3.30^BCD^	3.56^ABC^	0.08	<0.0001	0.0306	<0.0001
AP:ratio^5^	3.47^A^	2.99^B^	2.91^B^	2.20^F^	2.69^C^	2.48^D^	1.93^G^	2.35^E^	2.10^F^	0.06	<0.0001	<0.0001	<0.0001
Total bacteria	10.21^BC^	10.11^CD^	10.33^ABC^	10.47^A^	10.10^CD^	9.90^D^	10.44^AB^	10.16^BC^	10.32^ABC^	0.04	<0.0001	<0.0001	0.5301
Total archaea	8.78^A^	8.64^A^	8.74^A^	8.77^A^	8.61^AB^	8.59^AB^	8.71^A^	8.74^A^	8.39^B^	0.03	<0.0001	<0.0001	0.5642
Archaea (%)^6^	3.73^AB^	3.68^AB^	2.67^BCDE^	2.01^CDE^	3.26^BCD^	4.94^A^	1.92^DE^	3.63^ABC^	1.30^E^	0.20	<0.0001	0.1172	0.0094

*^1^IMI, imidazole at 100 mmol/L; PMSF, phenylmethylsulphonyl fluoride at 3 mmol/L; IOD, iodoacetamide at 0.5 mmol/L; SDS, sodium dodecyl sulfate at 1.44 mmol/L; ^2^3Mix, combination of IMI, PMSF, and IOD at the above concentrations; ^3^Treatment; ^4^Branched chain volatile fatty acids; ^5^Acetate: propionate ratio. ^6^% of archaea in total prokaryotes. Means (*n* = 8) followed by different superscripts in a row differ significantly (*P* < 0.05).*

The VFA profiles were affected by all the inhibitors, differently (Table 4). Total VFA concentration was decreased by all the treatments except PMSF and IOD. Except for IMI and IMI-PMSF, other treatments decreased the acetate molar proportion significantly. All the treatments increased the molar proportion of propionate, which corresponded to significantly decreased A:P ratio in all inhibitor treatments. All the inhibitor treatments decreased the molar proportion of butyrate except IOD that increased butyrate molar proportion significantly. IOD increased valerate molar proportion while IMI-PMSF decreased it. The molar proportion of branch-chain VFA (BCVFA) was significantly decreased by IOD, IMI-PMSF, and PMSF-IOD.

### Effect of the Lysozyme and Peptidase Inhibitors on the Predicted Functions of the Microbiota

**FIGURE 2:**
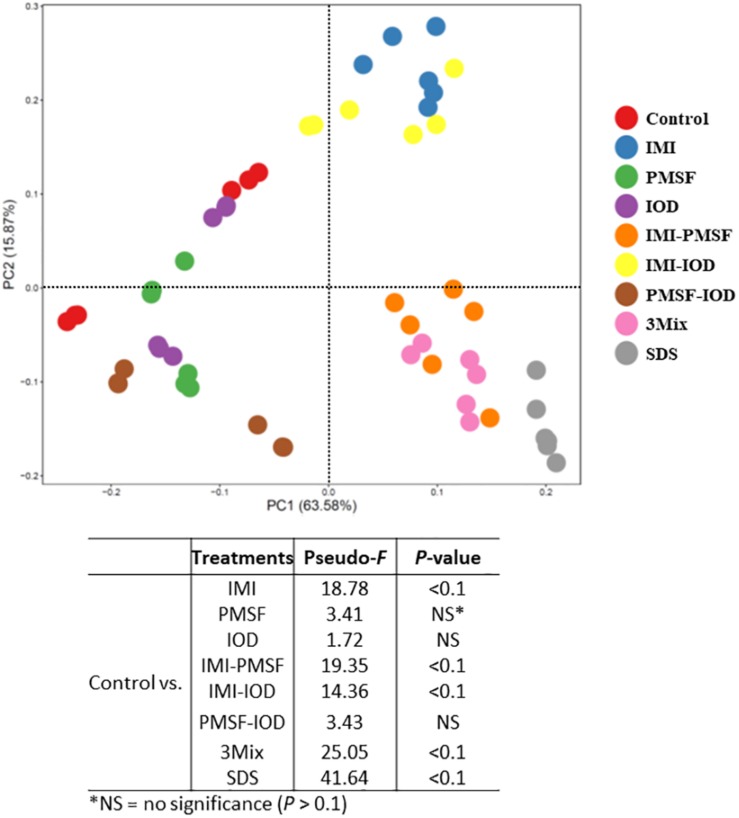
Principal components analysis (PCA) of the predicted functional features (based on KEGG orthologs and the CowPI database). PERMANOVA results of the pairwise comparison of the overall functional structures between control and each of inhibitor treatment was included.

All the IMI-containing treatments and SDS tended to affect the overall predicted functional features as assessed by PERMANOVA analysis (Figure 2). Correspondingly, the inhibitor treatments affected the KEGG ortholog (KO) groups involved in protein metabolism (Table 5). Amino acid metabolism was inhibited by all the inhibitor treatments. Lysosomal features were inhibited by IMI and its two- and three-way combinations. The PMSF-IOD combination and SDS also decreased lysosomal functional features. The ubiquitin system was not affected by any of the inhibitor treatments but significantly increased in relative abundance by SDS by nearly 2.44-fold. The relative abundance of peptidase features was decreased by all the inhibitor treatments except IOD. The inhibitor combinations led to a greater decrease of peptidase features, except for PMSF-IOD, compared to the individual inhibitors. The relative abundance of nitrogen-related metabolic functions was lowered significantly by SDS and IMI-containing treatments except for IMI-IOD, while other inhibitor treatments showing no effect. Overall, SDS showed the most inhibition, whereas IOD exhibited the least inhibition to the features involved in protein metabolism.

**TABLE 5:** Relative abundance (% of total) of CowPI predicted functional features related to protein metabolism at 24 h of incubation.

**Categories**	**Treatments^1^**									**SEM**	**Effects (*P*-values)**		
			
	**Control**	**IMI**	**PMSF**	**IOD**	**IMI-PMSF**	**IMI-IOD**	**PMSF-IOD**	**3Mix^2^**	**SDS**		**Trt^3^**	**Diet**	**T × D**
Level 2													
Amino acid metabolism	10.51^A^	9.67^D^	10.23^B^	10.31^B^	9.40^E^	9.84^CD^	9.98^C^	9.34^E^	9.03^F^	0.071	<0.0001	<0.0001	<0.0001
Level 3													
Lysosome	0.038^A^	0.018^DE^	0.034^AB^	0.036^AB^	0.024^CDE^	0.024^CD^	0.027^BC^	0.015^E^	0.006^F^	0.002	<0.0001	0.0106	0.0008
Ubiquitin system, ×10^–5^	6.687^B^	5.227^B^	0.975^B^	0.274^B^	8.243^AB^	4.912^B^	2.905^B^	9.039^AB^	16.342^A^	1.156	0.0001	0.0027	<0.0001
Peptidases	2.32^A^	2.27^BC^	2.24^CD^	2.29^AB^	2.14^E^	2.23^D^	2.23^D^	2.09^F^	2.04^G^	0.013	<0.0001	0.0590	<0.0001
Nitrogen metabolism	0.64^AB^	0.58^C^	0.66^A^	0.62^B^	0.57^C^	0.64^AB^	0.63^AB^	0.55^C^	0.56^C^	0.007	<0.0001	<0.0001	<0.0001

*^1^IMI, imidazole at 100 mmol/L; PMSF, phenylmethylsulphonyl fluoride at 3 mmol/L; IOD, iodoacetamide at 0.5 mmol/L; SDS, sodium dodecyl sulfate at 1.44 mmol/L. ^2^3Mix, combination of three inhibitors at the above concentrations. ^3^Treatment. Means (*n* = 6) followed by different superscripts in a row differ significantly (*P* < 0.05).*

**FIGURE 3:**
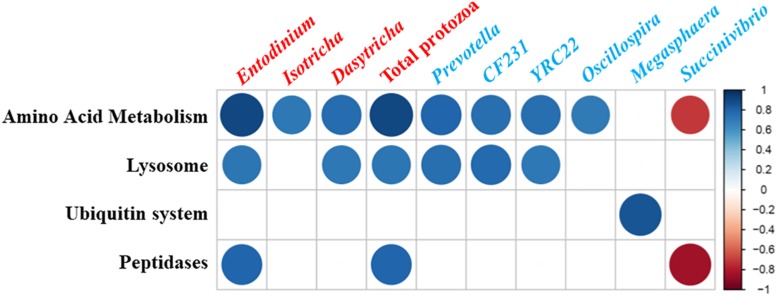
Correlations between microbial taxa [log-transformed counts of protozoal genera (labeled in red) and relative abundances of bacteria (labeled in blue) were used] and relative frequency of CowPI-predicted functional features related to protein metabolisms in the *in vitro* cultures. Only the strong correlations (Pearson correlation coefficient, |*r*| > 0.8; *P* < 0.05) were shown. The size and intensity of the color of each circle indicate the degree of the correlation coefficient based on the color key on the right side.

Strong correlations (Pearson correlation coefficient, | *r*| > 0.8, *P* < 0.05) were detected between some microbial taxa and the predicted functions related to protein metabolism (Figure 3). Total protozoal counts and the counts of three protozoal genera showed a positive correlation with amino acid metabolism. Among the enumerated protozoal genera, *Entodinium* was positively correlated with lysosomal and peptidase features while *Dasytricha* showed a positive correlation with lysosomal features. Positive correlation with amino acid metabolism and lysosomal features was found for *Prevotella*, *CF231* (a candidate genus in *Paraprevotellaceae*), and *YRC22* (another candidate genus in *Paraprevotellaceae*). *Succinivibrio* was negatively correlated with amino acid metabolism and peptidase features.

## Discussion

Rumen protozoa depend on live bacteria for nutrients essential for their survival and growth (Park et al., 2017), and their digestive enzymes including lysozyme and peptidases are required to digest the engulf bacteria (Bonhomme, 1990; Morgavi et al., 1996). However, their bacterivory causes intraruminal nitrogen recycling, resulting in lowered nitrogen utilization efficiency in ruminant animals (Kayouli et al., 1986; Belanche et al., 2011). Rumen protozoa also contribute to deaminase activity (Wallace et al., 1997). Thus, specific inhibition of the activities of lysozyme and peptidases of rumen protozoa is conceptually a sound approach to inhibit their growth and thereby decreasing nitrogen recycling in the rumen microbiome. The results of the present study demonstrated the promising potential of this new approach.

We collected rumen fluids from two cows fed different diets (high concentrate vs. hay only) because different diets can result in different protozoal composition. As expected, the rumen of both cows was dominated by *Entodinium* followed with other genera at low relative abundance. Thus, the protozoal composition in both cows was representative of that in dairy cows. The *in vitro* cultures were fed the same protozoal feed, which allows robust growth of both starch-preferring and cellulolytic rumen protozoa in *in vitro* monocultures (Park and Yu, 2018a). High numbers of live protozoa cells were achieved in control that contained no inhibitors. The absolute abundance of total bacteria and archaea was similar between the controls and all the inhibitor treatments, indicating the lack of overall inhibition to the prokaryotic populations. Therefore, the difference in protozoal counts between the control and the inhibitor treatments can be solely attributed to the impact of inhibitor treatments. It should be noted that the inhibition of rumen protozoa did not correspond to a significant increase in the abundance of total bacteria at 24 h incubation. One plausible explanation is that the growth of total bacteria might have been limited in the batch cultures that had limited nutrients and accumulated metabolites.

Different inhibitors inhibited different protozoal genera to a different extent. Among the inhibitors tested in this study, IMI was the most effective inhibitor regardless of the rumen protozoal genera. In a previous study, 1,2-dimethyl-5-nitroimidazole, a derivative of IMI was shown to be inhibitory against both holotrichs and entodiniomorphs (Clarke and Reid, 1969). These results corroborate the essentiality of lysozyme for rumen protozoa. The cysteine peptidase inhibitor, IOD, mainly inhibited large entodiniomorphs, but IMI and PMSF inhibited most of the identified rumen protozoa. Except IOD, all the inhibitor treatments also reduced all the eight detected protozoal genera in a time-dependent manner, suggesting that the inhibitors probably have caused starvation rather than direct toxicity. The varying potencies of the inhibitors reflect different susceptibilities of the protozoal genera toward the inhibitors, but the mechanisms remain to be elucidated. All the three inhibitors nearly eliminated *E. caudatum* in the laboratory monoculture of *E. caudatum* (Park et al., 2019), but in the present study, much less inhibition, including inhibition to the genus *Entodinium*, was achieved, especially by individual inhibitors. The *in vitro* cultures of the present study differed from the *E. caudatum* monoculture in prokaryotic microbiota (complex vs. relatively simple), initial protozoal density (appx. fivefold higher in the *in vitro* cultures of the present study), and diversity (one vs. eight genera). One or both of these differences may explain the decreased inhibition observed in the present study.

Among the two-way combinations of lysozyme and peptidase inhibitors, IMI-IOD showed the least synergistic effect on the rumen protozoa, with no additive inhibition being observed to any of the protozoal genera. The other two two-way combinations (i.e., IMI-PMSF and PMSF-IOD) mostly had synergistic inhibition. However, these two latter combinations had no additive inhibition toward *Polyplastron*. Nonetheless, the inhibitors in combinations were more inhibitory than individual inhibitors, and they may be further evaluated together *in vivo*.

A significant decrease in ruminal ammonia concentration corresponded to defaunation (Newbold et al., 2015). In the present study, however, IMI and SDS resulted in the greatest inhibition to total protozoa, but they did not decrease ammoniagenesis. On the other hand, the cysteine peptidase inhibitor (IOD) decreased total protozoa the least, but it reduced the ammonia concentration the greatest, and the serine peptidase inhibitor (PMSF) only decreased ammoniagenesis when used in two- and three-way combinations. These results suggest several possibilities. First, increased bacterial proteolysis in batch cultures (due to autolysis resulted from lack of continuous replenishment of nutrients and removal of metabolites) might have compensated the decreased ammoniagenesis by protozoa. Second, cysteine peptidases probably play a larger role than serine peptidases in ammoniagenesis by rumen protozoa. This postulation is corroborated by a previous study (Forsberg et al., 1984) and cysteine peptidases being the major type of bacterial peptidases in the rumen (Kopecny and Wallace, 1982; McSweeney and Mackie, 2012). The large increase in relative abundance of *Acidaminococcus*, a genus of potential hyper-ammonia producing bacteria (HAB) (Cook et al., 1994) which was undetectable in the control but detectable in the IMI-containing inhibitor treatments and SDS treatments, corroborates the above premise. Thirds, the peptidase inhibitors probably had also inhibited bacterial peptidases as suggested previously (Forsberg et al., 1984). In the control culture, *Ruminobacter* had a >15% relative abundance. In addition to its strong proteolytic activity (Akkada and Blackburn, 1963; Goodfellow et al., 2005), *Ruminobacter amylophilus*, the representative species of this genus, has cell-associated serine peptidase as the major peptidase (Wallace and Brammall, 1985). The lower predominance of this genus in the inhibitor treatments containing PMSF, though not detected by ANCOM as a differentially abundant taxon, supports the efficacy of PMSF on its target and the decreased ammonia concentration in those PMSF-containing combinatorial inhibitor treatments. Future research using protozoa-free and protozoa-containing rumen microbiota can help determine if and to what extent peptidase inhibitors can inhibit bacteria ammoniagenesis. Nonetheless, peptidase inhibitors are probably more effective than lysozyme inhibitors in lowering ammoniagenesis, and inhibition of both lysozyme and peptidases, particularly inhibition of cysteine peptidases, is probably more effective in reducing ammoniagenesis in the rumen.

Both DMD and NDFD were decreased by IMI and the IMI-containing inhibitor treatments in the *in vitro* cultures. In the previous study using *E. caudatum* monoculture, DMD and NDFD were also decreased by IMI-containing inhibitor treatments at least numerically (Park et al., 2019). The IMI and IMI-containing treatments inhibited protozoa to a greater magnitude than the other inhibitors. The decrease in feed digestion in defaunated animals (Newbold et al., 2015) seems consistent with the larger DMD and NDFD decrease in the IMI and the IMI-containing treatments, but it remains to be determined if IMI can directly inhibit feed digestion. *Fibrobacter* was greatly decreased by the treatments that significantly lowered NDFD. A positive correlation between *Fibrobacter* and holotrichs was reported in a recent global rumen microbiome survey (Henderson et al., 2015), and a decrease in *Fibrobacter* abundance was also noticed after defaunation (Ozutsumi et al., 2006). The moderate protozoal inhibition by PMSF and IOD did not correspond to a significant decrease in DMD or NDFD, which is consistent with the previous study using *E. caudatum* monoculture (Park et al., 2019). Future research can help determine if the inhibitors, especially IMI, directly inhibit *Fibrobacter* and other known fibrolytic bacteria such as species of *Ruminococcus*.

Rumen protozoa can affect the VFA profiles both directly and indirectly. Decreased butyrate in the inhibitor treatments except IOD is consistent with the finding in defaunated animals (Eugène et al., 2004) and *in vitro* rumen cultures when rumen protozoa were inhibited by saponin (Patra and Saxena, 2009; Jayanegara et al., 2014; Ramos-Morales et al., 2017). Protozoa itself produce butyrate in the rumen (Coleman, 1975; Jouany, 1991). Because the abundance of major butyrate producers including *Butyrivibrio* and *Pseudobutyrivibrio* did not change with the exception of increase of the latter genus by IOD, protozoa inhibition was probably the major reason for the decreased butyrate proportion in the inhibitor treatments. Opposite trends of acetate and propionate molar proportion resulted in decreased A:P ratio in most of the inhibitor treatments, which is consistent with decreased A:P ratio observed in defaunated sheep fed high-concentrate diet (Mendoza et al., 1993). A similar VFA profile shift was also reported in a recent meta-analysis of the defaunation effect on rumen fermentation (Li et al., 2018). The bacterial population shifts in the inhibitor treatments could also contribute to the shifted VFA profile. The decrease of rumen protozoa in the inhibitor treatments corresponded with increase of amylolytic and saccharolytic bacteria, such as *Treponema*, *Streptococcus*, and *Succinivibrio*, all of which are known sugar-fermenting bacteria in the rumen (Stanton and Canale-Parola, 1980; Patterson and Hespell, 1985; O’Herrin and Kenealy, 1993; Nagaraja, 2016). The negative correlation between rumen protozoa and amylolytic bacteria has been previously noted (Arakaki et al., 1994; Bełżecki and Michałowski, 2005), and the increase of these bacteria could be attributed to the decreased predation and the lack of competition for starch from rumen protozoa (Eadie and Mann, 1970; Kurihara et al., 1978).

The overall microbiota was shifted differently by the different inhibitor treatments. The compositional alterations might have stemmed from direct and/or indirect effects. Rumen protozoa have multiple interactions with prokaryotes, such as predation, symbiosis (both endosymbiosis and ectosymbiosis), and commensalism (e.g., cross-feeding between protozoa and methanogens and amino-acid fermenters) (Williams and Coleman, 1992; Lloyd et al., 1996; Park and Yu, 2018b). The different inhibitor treatments might have caused different alterations of the prokaryotic microbiota indirectly by inhibiting the different protozoa to different extents. However, the inhibitors might also have directly shifted some of the prokaryotic populations. Future research can help evaluate likely direct effects of the inhibitors tested in the present study on the rumen prokaryotic microbiota by including protozoa-free *in vitro* cultures.

It is interesting to note that the inhibitors affected most of the alpha diversity measurements of the prokaryotic microbiota to a greater magnitude in the *in vitro* cultures of the dairy ration-fed cow than in the *in vitro* cultures of the hay-fed cow. Given the difference in the prokaryotic microbiota of the inocula between the dairy ration-fed and the hay-fed rumen (e.g., lower species richness and Faith’s phylogenetic diversity in the inoculum of the hay-fed rumen, data not shown), the “background” prokaryotic microbiota of rumen fluid inoculum seem to be a factor that can affect the effect of the inhibitors. Moreover, recent studies revealed a negative association between rumen microbiota diversity and feed efficiency in ruminants (Shabat et al., 2016; Li and Guan, 2017). A meta-analysis also revealed that defaunation lowered dry matter intake, heat-production, and ammonia concentrations but increased average daily gain, duodenal nitrogen flow, energy efficiency for fattening in addition to methane reduction (Newbold et al., 2015). It is speculative, but inhibition of rumen protozoa can potentially enhance the overall feed efficiency in addition to the expected improvement of nitrogen efficiency and decrease in methane emission from ruminants.

Comparison of the co-occurrence and mutual-exclusion networks of major prokaryotic taxa showed clear differences in co-occurrence patterns among the control and the inhibitor treatments. The alteration of co-occurrence and mutual-exclusion is consistent with the shifts of the prokaryotic microbiota and its lowered alpha-diversity measurements. The inhibitors might have altered the interactions not only between protozoa and prokaryotes but also among different prokaryotes. Such alterations may be attributed to changes in predation pressure from decreased protozoa and or subsequent alteration of ecological interactions among some of the microbes. However, the data of the present study do not allow distinguishability of these two possibilities.

Strong positive correlations were detected between *Entodinium* counts and lysosome and peptidase functions. This is consistent with *Entodinium* being considered as the principal culprit of microbial nitrogen recycling due to its abundance and high bacterivory activity (Coleman and Sandford, 1979; De La Fuente et al., 2011; Belanche et al., 2012). Previous research also observed the greater defaunation effect when animals were fed a mixed diet (Belanche et al., 2011) and when starch-preferring *Entodinium* accounted for nearly all the protozoa. Given the greater effect being observed in the *in vitro* cultures of the dairy ration-fed cow, protozoa inhibition can probably result in more improvement in animals fed mixed diets, which typically have a much greater total protozoal population than in animals fed forage.

Taken together, the lysozyme inhibitor at the tested concentration achieved the greatest inhibition of rumen protozoa, and it also decreased feed digestibility. However, inhibition of rumen protozoa by the peptidase inhibitors did not adversely decrease feed digestion or fermentation although it shifted some of the prokaryotic populations and fermentation profiles. The peptidase inhibitors might inhibit peptidases of both protozoa and bacterial origin. Cysteine peptidase inhibitors may be more effective than serine peptidase inhibitors in inhibiting ammoniagenesis by rumen microbiome. Future research is needed to determine if these inhibitors also directly inhibit some of the rumen prokaryotes, particularly cellulolytic bacteria, amylolytic bacteria, and proteolytic bacteria. *In vivo* studies are also needed to verify if these inhibitors can be effective when fed to animals. Of course, the tested inhibitors are chemical inhibitors, and their toxicity to host animals needs to be determined first. Natural compounds can be screened and explored for animal applications. Overall, specific inhibition of lysozyme and peptidases may represent a new approach to effectively improve feed efficiency, particularly nitrogen utilization efficiency, and decrease methane emission from ruminants.

## Data Availability Statement

The datasets generated for this study can be found in the NCBI Sequence Read Archive under BioProject PRJNA523838.

## Author Contributions

TP and ZY conceived the experiment and wrote the manuscript. TP and HM conducted the experiment. TP analyzed the fermentation and microbial sequencing data. TP, HM, and ZY reviewed the manuscript and read and approved the final manuscript.

## Conflict of Interest

The authors declare that the research was conducted in the absence of any commercial or financial relationships that could be construed as a potential conflict of interest.
