# Supplementary material for: Inhibition of Rumen Protozoa by Specific Inhibitors of Lysozyme and Peptidases in vitro
Source: Front Microbiol. 2019 Dec 6;10:2822. doi: 10.3389/fmicb.2019.02822 (PMC6908469; doi:10.3389/fmicb.2019.02822)
Supplement: Supplementary file 1 [file Data_Sheet_1.DOCX]

Supplementary Material

Inhibition of rumen protozoa by specific inhibitors of lysozyme and peptidases *in vitro*

Running title: Specific inhibitors of lysozyme and peptidases inhibit rumen protozoa

Tansol Park^1^, Huiling Mao,^1,2^ and Zhongtang Yu^1^*

^1^ Department of Animal Sciences, The Ohio State University, Columbus, OH, United States

^2^ College of Animal Science and Technology, Zhejiang A & F University, Lin’an, P. R. China

*** Correspondence:** Zhongtang Yu: [yu.226@osu.edu](mailto:yu.226@osu.edu)

**Supplementary Data**

**Figure S1.** Relative abundance of the major (A) phyla and (B) genera of prokaryotes (each representing >0.5% at least one of the treatments).

**Figure S2.** Co-occurrence and mutual-exclusion networks showing the correlations between major prokaryotic genera (each representing >0.5% at least one of treatments) in the *in vitro* cultures. Only the significant correlations (with adjusted *P*-value < 0.05) were shown. Blue and red lines indicate positive and negative association, respectively. Color legend showed the belonged phylum of each node

**Table S1.** DADA2 denoising statistics of the amplicon sequencing variants (at 24 h of incubation).

| Treatments^1^ | No. of input paired reads | Quality filtered reads | Denoised reads | Merged reads | Chimera filtered reads | Taxa-filtered^2^ |
| --- | --- | --- | --- | --- | --- | --- |
| Inoculum | 100,118 | 81,846 | 81,846 | 68,550 | 59,971 | 59,862 |
| Control | 95,978 | 79,471 | 79,471 | 67,633 | 59,984 | 59,914 |
| IMI | 74,397 | 63,658 | 63,658 | 57,091 | 49,267 | 49,143 |
| PMSF | 81,323 | 69,939 | 69,939 | 59,578 | 50,484 | 50,420 |
| IOD | 56,776 | 47,654 | 47,654 | 40,195 | 34,985 | 34,983 |
| IMI-PMSF | 52,137 | 44,845 | 44,845 | 41,225 | 35,995 | 35,900 |
| IMI-IOD | 48,360 | 41,947 | 41,947 | 37,219 | 31,008 | 30,921 |
| PMSF-IOD | 59,346 | 50,180 | 50,180 | 43,938 | 35,849 | 35,848 |
| 3Mix^3^ | 56,905 | 49,273 | 49,273 | 44,052 | 36,617 | 36,504 |
| SDS | 70,385 | 61,122 | 61,122 | 56,258 | 42,074 | 42,074 |
| SEM | 3,376 | 2,768 | 2,768 | 2,418 | 2,092 | 2,090 |

^1^ IMI, imidazole at 100 mmol/L; PMSF, phenylmethylsulphonyl fluoride at 3 mmol/L; IOD, iodoacetamide at 0.5 mmol/L; SDS, sodium dodecyl sulfate at 1.44 mmol/L.

^2^ Possible sequences of mitochondria and chloroplast were filtered out. Sequences that could not be assigned to Bacteria or Archaea were also excluded.

^3^ 3Mix, combination of three inhibitors at the above concentrations.

Each value represented the mean of six replicates.
