# Supplementary figures and images for: Inhibition of Rumen Protozoa by Specific Inhibitors of Lysozyme and Peptidases in vitro
Source: Front Microbiol. 2019 Dec 6;10:2822. doi: 10.3389/fmicb.2019.02822 (PMC6908469; doi:10.3389/fmicb.2019.02822)

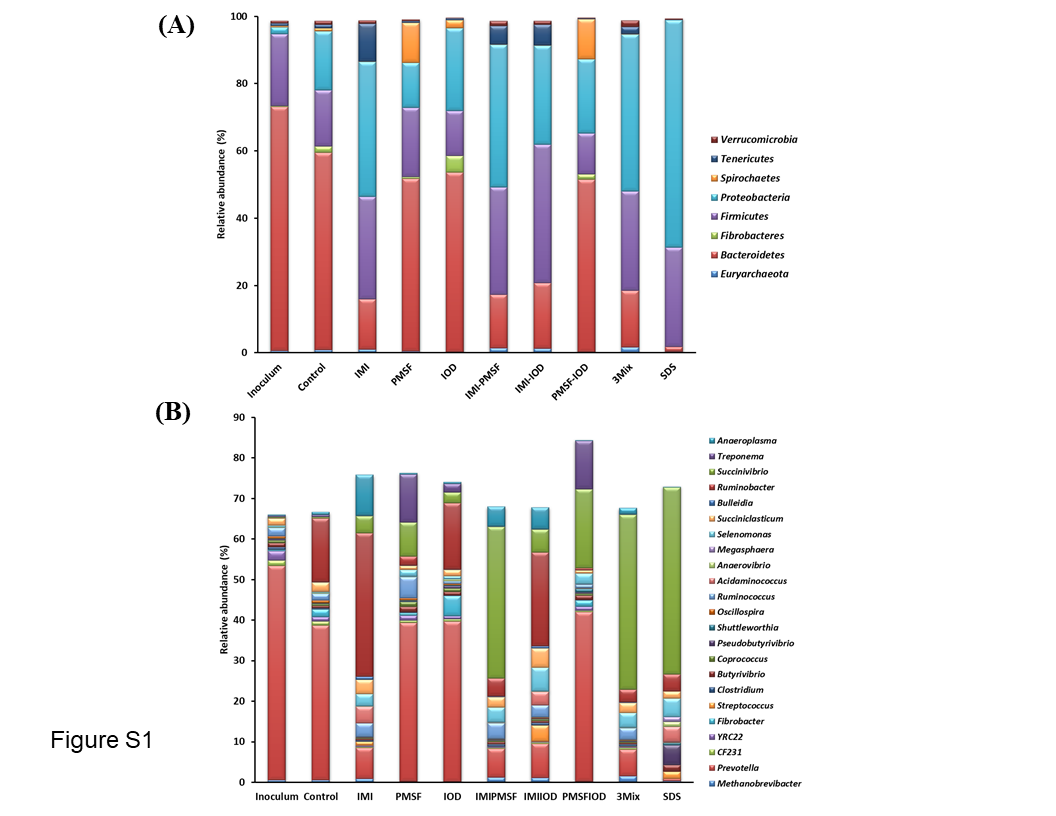

Supplement: Supplementary file 2 [file Image_1.TIF]

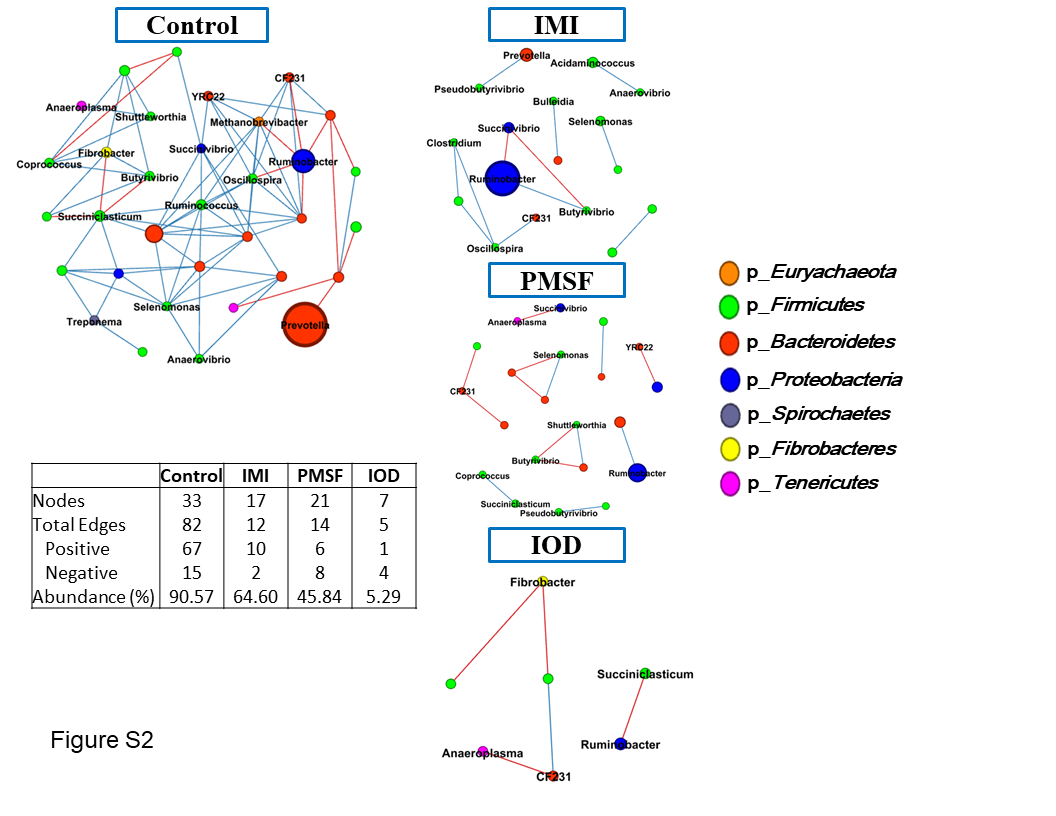

Supplement: Supplementary file 3 [file Image_2.TIF]
